# Supplementary material for: A Global Screen for Assembly State Changes of the Mitotic Proteome by SEC-SWATH-MS
Source: Cell Syst. 2020 Feb 26;10(2):133–155.e6. doi: 10.1016/j.cels.2020.01.001 (PMC7042714; doi:10.1016/j.cels.2020.01.001)

Q9Y320 | TMX2_HUMAN | TMX2 TXNDC14 CGI−31 My009 PIG26 PSEC0045 UNQ237/PRO270
Monomer MW [kDa]: 34.038 Monomer expected elution fraction: 50

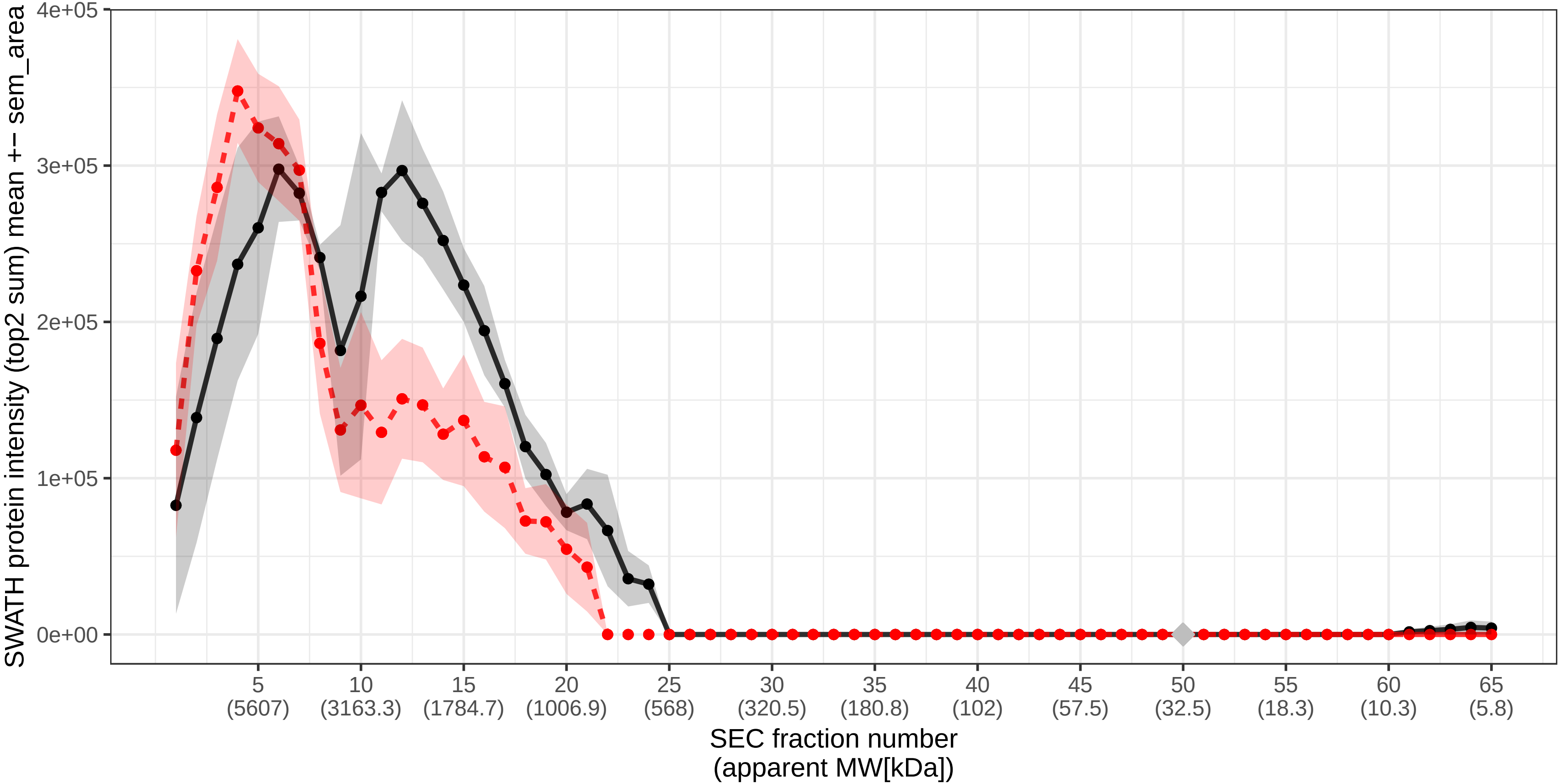

Supplement: Data S1. SEC-SWATH-MS Protein Chromatograms, Related to Figure 1 [file mmc6.zip › SECchrom_Q9Y320_TMX2_HUMAN_TMX2_TXNDC14_CGI-31_My009_PIG26_.pdf]
